# Hydroxysafflor Yellow A Phytosomes Administered via Intervaginal Space Injection Ameliorate Pulmonary Fibrosis in Mice

**DOI:** 10.3390/ph15111394

**Published:** 2022-11-12

**Authors:** Tingting Li, Dong Han, Zhongxian Li, Mengqi Qiu, Yuting Zhu, Kai Li, Jiawei Xiang, Huizhen Sun, Yahong Shi, Tun Yan, Xiaoli Shi, Qiang Zhang

**Affiliations:** 1 College of Life Sciences, Bejing University of Chinese Medicine, Beijing 100029, China; 2 Hebei Key Lab of Nano-Biotechnology, Hebei Key Lab of Applied Chemistry, Yanshan University, Qinhuangdao 066004, China; 3 CAS Center for Excellence in Nanoscience, National Center for Nanoscience and Technology, Beijing 100190, China; 4 School of Future Technology, University of Chinese Academy of Sciences, Beijing 100049, China

**Keywords:** intervaginal space injection, pulmonary fibrosis, hydroxysafflor yellow A, phytosome, interstitial treatment

## Abstract

Idiopathic pulmonary fibrosis is a fatal interstitial disease characterized by fibroblast proliferation and differentiation and abnormal accumulation of extracellular matrix, with high mortality and an increasing annual incidence. Since few drugs are available for the treatment of pulmonary fibrosis, there is an urgent need for high-efficiency therapeutic drugs and treatment methods to reduce the mortality associated with pulmonary fibrosis. The interstitium, a highly efficient transportation system that pervades the body, plays an important role in the occurrence and development of disease, and can be used as a new route for disease diagnosis and treatment. In this study, we evaluated the administration of hydroxysafflor yellow A phytosomes via intervaginal space injection (ISI) as an anti-pulmonary fibrosis treatment. Our results show that this therapeutic strategy blocked the activation of p38 protein in the MAPK-p38 signaling pathway and inhibited the expression of Smad3 protein in the TGF-β/Smad signaling pathway, thereby reducing secretion of related inflammatory factors, deposition of collagen in the lungs of mice, and destruction of the alveolar structure. Use of ISI in the treatment of pulmonary fibrosis provides a potential novel therapeutic modality for the disease.

## 1. Introduction

Idiopathic pulmonary fibrosis is an irreversible and fatal interstitial lung disease with a high mortality rate, increasing annual incidence, and extremely poor prognosis for patients, with a survival time of 3–5 years after diagnosis [1]. The current coronavirus disease 2019 (COVID-19) pandemic has been gradually controlled, but the consequent emergence of complications, such as pulmonary fibrosis, has caused widespread concern. According to the World Health Organization, more than 562 million people have been infected with COVID-19 worldwide and more than 6.3 million people have died [2]. Most mild and moderate cases of COVID-19 can recover, but some severely ill patients still have persistent hypoxemia, and chest imaging shows the characteristic pattern of fibrosis [3]. Complications of pulmonary fibrosis can occur in some patients, even after discharge from the hospital [4,5,6,7]. Therefore, development of efficient therapeutic drugs and treatment modalities to reduce pulmonary fibrosis is the key to effectively and completely curing patients with COVID-19. The process of pulmonary fibrosis formation can be considered an uncontrolled wound-healing response, which is ultimately manifested as massive proliferation and aggregation of myofibroblasts and excessive deposition of extracellular matrix in the alveoli and interstitium of the lung, leading to a reduction in lung volume, thickening of alveoli septa, and a loss of lung tissue elasticity [8]. However, the pathogenesis of fibrosis is not completely clear, and the only antifibrotic drugs currently approved for marketing are pirfenidone and nintedanib, which have narrow indications, limited duration of administration, and some side effects. These drugs are not widely used in clinical practice [9]. Therefore, there is an urgent need for efficient therapeutic drugs and treatment methods to improve the current status of pulmonary fibrosis treatment.

The interstitium is composed of a fibrous network and matrix, which is located between cells, substantive functional tissues, and organs, forming a multi-level network structure from a loose fibrous network to a dense fibrous stroma structure, making the body a flat and connected organic whole [10,11]. The structure and composition of the interstitium in different organs vary but are similar to some extent, consisting of flowing interstitial fluid and a complex network of collagen bundles [12]. In addition to nutritional and mechanical support functions, the interstitial system has efficient connectivity pathways, which can serve as transportation channels for cancer cells, immune cells, and microorganisms, and is involved in pathophysiological processes, such as cancer cell metastasis, fibrosis, and edema [12,13]. These channels may be a potential conduit for damaging substances and pro-fibrotic signaling molecules, whereas interstitial cells may be precursors of myofibroblasts and play an important role in the formation of fibrosis. Therefore, the interstitium may play a crucial role in the occurrence and development of pulmonary fibrosis. However, the importance of the interstitium is neglected in the diagnosis and treatment of the disease. The combination of interstitial pathways with disease prevention and treatment will bring new technical means and application methods to scientific research and clinical practice. Intervaginal space injection (ISI) can deliver nanoparticles to various organs without relying on blood circulation, confirming that the interstitial system can serve as a long-range and efficient transportation pathway, providing a potentially efficient option for targeted drug delivery [14,15,16,17]. Therefore, the introduction of ISI as a novel drug delivery method in the treatment of pulmonary fibrosis may play a crucial role in the behavior of interstitial myofibroblasts and in improving the interstitial microenvironment.

Hydroxysafflor yellow A (HSYA), a compound with a monochalcone glycoside structure, is the most effective water-soluble ingredient of safflower that has been widely used in the treatment of cardiovascular diseases because of its effective anticoagulant, anti-myocardial ischemic, and vasorelaxant effects [18,19,20,21]. Studies have shown that HSYA inhibits lipopolysaccharide-induced proliferation, migration, and invasion of non-small-cell lung cancer cells by inhibiting the PI3K and AKT signaling pathways [22] and TGF-β1-induced activation of human lung fibroblasts in vitro [23]. HSYA has been shown to have anti-inflammatory, antioxidant, and apoptosis-inhibiting effects [24], and it has potential applications in the treatment of dementia, Parkinson’s disease, and tumors.

Herein, hydroxysafflor yellow A phytosome (HYAP), as an anti-pulmonary fibrosis therapeutic agent, was prepared to improve the lipid solubility and bioavailability of HSYA, to treat bleomycin-induced pulmonary fibrosis in mice by ISI. This therapeutic strategy alleviates the severity of pulmonary fibrosis, elaborates the mechanism of interstitial treatment, and provides a novel therapeutic approach for the treatment of pulmonary fibrosis.

## 2. Results

### 2.1. Characterization of HYAP

**Figure 1:**
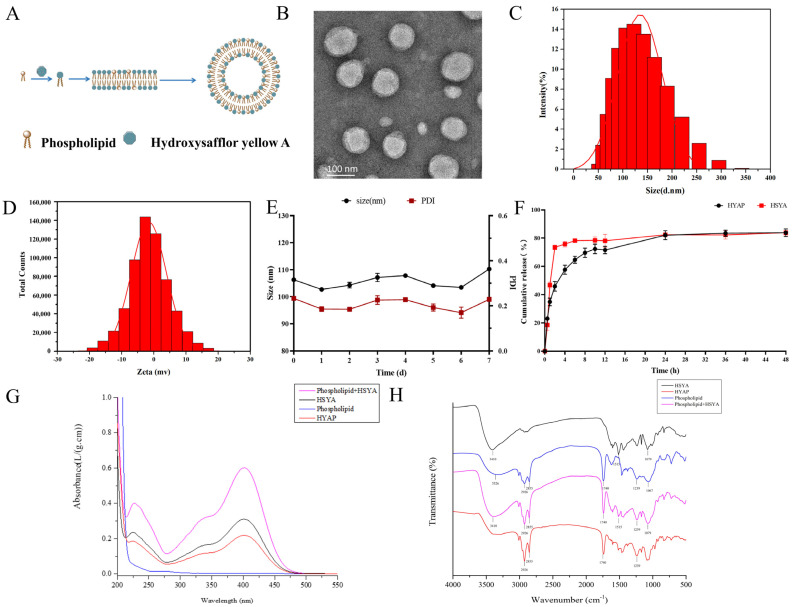
Preparation and characterization of HYAP. (**A**) Schematic diagram of the preparation of HYAP. (**B**) Morphology of HYAP by TEM. Scale bar: 100 nm. (**C**) Size distribution of HYAP by DLS. (**D**) Zeta-potential of HYAP. (**E**) Size and PDI changes of HYAP in seven days. (**F**) Cumulative release rate of HYAP and HSYA in vitro by UV. (**G**) Ultraviolet spectroscopy analysis of HSYA, phospholipids, mixture of HSYA and phospholipids, and HYAP. (**H**) Fourier transform infrared spectroscopy analysis of HSYA, phospholipids, mixture of HSYA and phospholipids, and HYAP.

HSYA has poor lipid solubility, which results in low oral bioavailability. Hence, we prepared the HYAP to increase its lipid solubility and prolong its release time to improve its efficacy. Phytosomes are similar to liposomes in many ways: both can alter the physicochemical properties of the parent drug and improve the bioavailability of the drug, but they differ greatly in their principles of formation. A liposome is a drug encapsulated in a closed vesicle formed by a phospholipid, and the drug is either free in the solution within the vesicle or dispersed between multiple membranes of the phospholipid. In contrast, phytosomes are more stable compounds or complexes formed by charge migration between the drug and the phospholipid. The oxygen atom in the hydroxyl group on the phospholipid atom in the phospholipid structure has a strong tendency to gain electrons, while the nitrogen atom has a strong tendency to lose electrons, so under certain conditions, it can generate complexes with drug molecules of a certain structure. In the process of HYAP formation, mainly HSYA acts by forming hydrogen bonds or van der Waals forces with phospholipid molecules (Figure 1A). As for water-soluble drugs, making phytosomes can improve the encapsulation rate of the drug compared to liposomes, so we chose to prepare HYAP to improve the lipid solubility of free HSYA and to improve its bioavailability.

The prepared HYAP was characterized by transmission electron microscopy (TEM). HYAP was uniformly spherical, and the sizes were mainly concentrated around 101.67 nm (Figure 1B). The hydration diameter measured by dynamic light scattering (DLS) was mainly concentrated at 106.3 nm (Figure 1C), which is consistent with the TEM results. PDI was 0.23, indicating that the size distribution was relatively uniform. The zeta-potential was approximately -1.37 mv (Figure 1D). In addition, we evaluated the average size and PDI changes of the nanoparticles for 7 days, and no significant size changes were found during the 7 days and the PDI remained small, which proves that the nanoparticles have a uniform size distribution (Figure 1E).

The compounding rate of HYAP was 91.4% and the encapsulation rate was 57.86%, as measured by the ultraviolet spectrophotometer (UV). The cumulative release rate of HSYA reached 73.48% at 2 h, 75.85% at 4 h, and leveled off around 6 h, with a cumulative release rate of 83.84% at 48 h. The cumulative release rate of HYAP was 45.88% at 2 h, followed by a rapid release period for the first 4 h, with a cumulative release rate of 57.65% at 4 h, followed by a slow release, which leveled off at 24 h. The cumulative release rate was 83.88% after 48 h (Figure 1F). This shows that HYAP can significantly slow down the release of the drug and maintain a long drug release cycle.

We then analyzed the physicochemical properties of the HYAP by UV and Fourier transform infrared spectrometer (FT-IR). As shown in Figure 1, HSYA, mixture of HSYA and phospholipids, and HYAP have the maximum absorption peak at 403 nm, and the spectra are similar. The peak is the maximum absorption peak of HSYA, while phospholipids have no absorption peak at 403 nm (Figure 1G), indicating that HSYA and phospholipids do not form new chromophores in the compounding process and phospholipids at 403 nm do not interfere with the detection of HSYA.

The characteristic absorption peaks of HSYA were 3400 cm^-1^ (-OH), 1515 cm^-1^ (C=O), and 1079 cm^-1^ (C-O), and the characteristic absorption peaks of phospholipids were 3526 cm^-1^ (-OH), 2926 and 2855 cm^-1^ (C-H), 1740 cm^-1^ (C=O), 1239 cm^-1^ (P=O), and 1067 cm^-1^ (P-O-C) (Figure 1H). The characteristic absorption peaks on FT-IR of the mixture are basically the superposition of the main peaks of both, indicating that there is no interaction between the two in the mixture. The FT-IR spectrum of HYAP is different. HSYA has a sharp absorption peak at 3410 cm^-1^ due to the hydroxyl structure, which also appears in the mixture. In HYAP, the peak disappeared and only showed a broad peak at 3000–3500 cm^-1^, similar to that of phospholipids, indicating that the hydroxyl group of HSYA in the phytosomes may have intermolecular forces with phospholipids. Additionally, in the HYAP spectrum, the size and shape of the peak at 1515 cm^−1^ changed somewhat. In addition, it can be speculated that there may be hydrogen bonds or van der Waals forces formed between HSYA and phospholipids from the small changes in the characteristic peaks of the fingerprint region. 

### 2.2. Histomorphological Recovery after Interstitial Treatment 

**Figure 2:**
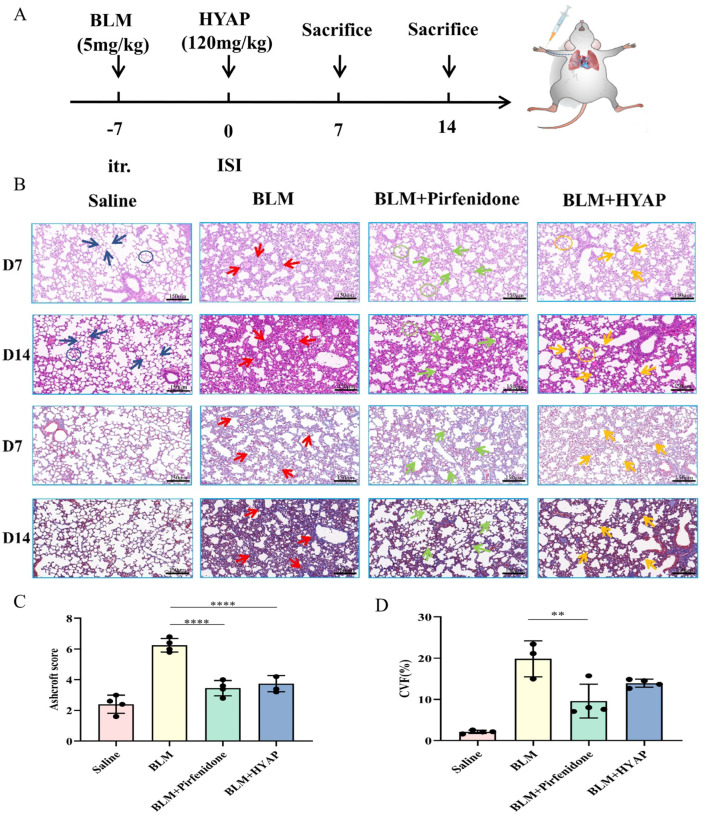
Histomorphological changes in the lungs of treated mice. (**A**) Schematic diagram of model establishment and treatment. (**B**) HE (**top**) and Masson trichrome staining (**bottom**) of lung tissue after different treatments (*n* = 4). Blue arrows indicate the interval between normal pulmonary alveoli in the saline group, and blue circles indicate intact structure of pulmonary alveoli. Red arrows indicate the interalveolar septa in the BLM group that were significantly thickened, and blue collagen fibers were significantly more abundant. Green arrows indicate that the number of interalveolar septa reduced, and collagen deposition decreased in the BLM + pirfenidone group. The green circle indicates that the structure of pulmonary alveoli was restored. Yellow arrows indicate that the number of interalveolar septa was also significantly reduced in mice treated with HYAP, with a concomitant decrease in collagen deposition. Yellow circles indicate the intact structures of pulmonary alveoli. Scale bar: 150 μm. (**C**) HE staining in different groups after 14 days of treatment was evaluated using the Ashcroft scoring method (*n* = 4). The BLM group vs. the BLM + pirfenidone group, **** *p* < 0.0001, and the BLM group vs. the BLM + HYAP group, **** *p* < 0.0001. (**D**) The fraction of collagen volume of Masson trichrome staining after 14 days of treatment was calculated using Image J software (*n* = 4). The BLM group vs. the BLM + pirfenidone group, ** *p* < 0.01.

The pulmonary fibrosis model was established by intratracheal injection of BLM, and HYAP was administered by ISI for 14 days after the successful establishment of pulmonary fibrosis on day 7 (Figure 2A). To compare the degree of fibrosis and recovery in each group of mice, we performed HE and Masson trichrome staining to observe the inflammatory response, fibrotic changes, and destruction of lung tissues (Figure 2B).

HE staining showed that the alveolar structure in the saline group was clear and intact, the alveolar septum was not thickened, and no inflammatory cell infiltration was observed, whereas the alveolar structure in the model group was seriously damaged, fibrous tissue proliferated, and the septum between alveoli was significantly thickened. Compared with the BLM group, the BLM + pirfenidone and BLM + HYAP groups showed greater improvement in the degree of lung tissue destruction, thinner alveolar septum, and complete recovery of alveolar structure, and this phenomenon became more obvious with the increase in days of administration (Figure 2B). The Ashcroft scores of the BLM + pirfenidone and BLM + HYAP groups decreased significantly (Figure 2C), compared with the BLM group, showing that the degree of pulmonary fibrosis was reduced.

Masson trichrome staining showed that only a small amount of collagen was deposited in the lung tissue of the mice in the saline group, whereas a large amount of blue collagen was diffusely deposited in the interstitium of the lung tissue of mice in the BLM group. Compared with the BLM group, the extent of lung tissue lesions was reduced in both the BLM + pirfenidone and BLM + HYAP groups, and the blue collagen deposition was significantly reduced (Figure 2B). The fraction of collagen volume in Masson trichrome staining was calculated using the Image J software. After treatment, the fraction of collagen in the lungs of the BLM + HYAP group was lower than that in the BLM group (Figure 2D), demonstrating that lung fibrosis in mice caused by BLM could be improved by HYAP after interstitial treatment.

### 2.3. Micro-CT Imaging Improvement In Vivo after Interstitial Treatment 

**Figure 3:**
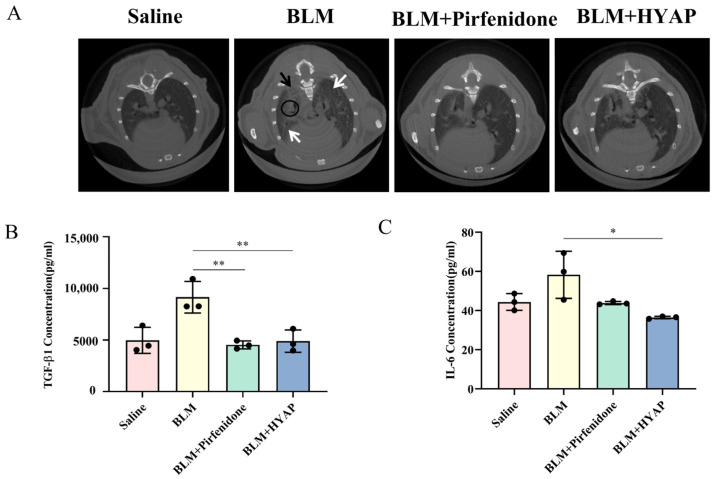
Micro-CT imaging analysis and secretion levels of inflammatory factors in treated mice after 14 days of treatment. (**A**) CT imaging of mice after treatments (*n* = 3). Black circles indicate a large number of high-density shadows that replaced the original lung parenchyma. White arrows indicate ground glass shadows. Black arrows indicate honeycomb shadows. (**B**,**C**) Secretion levels of TGF-β1 and IL-6 in mice plasma after treatments were determined using ELISA (*n* = 3). ** *p* < 0.01, * *p* < 0.05.

To assess the disease development in mice in each group after model establishment and administration, we performed computed tomography (CT) imaging analysis (Figure 3A). The grayscale of the micro-CT image reflects the change in material density. In CT images, high-density bone was reflected as white and muscle and blood vessels were reflected as light gray, whereas the lung parenchyma was mainly composed of air-filled pulmonary alveoli and soft alveolar walls, which are shown in dark gray. In the saline group, the lung parenchyma was black, and the distribution of gray airways can be seen in the CT images. In the BLM group, a large number of high-density shadows were observed instead of the original part of the lung parenchyma, with ground glass and honeycomb shadows, indicating a severe degree of fibrosis and proving the successful establishment of a fibrosis model. Mice in the BLM + pirfenidone and BLM + HYAP groups showed significant improvement in the ground glass shadow compared with those in the BLM group (Figure 3A), and parts of the black lung parenchyma were completely restored, whereas no obvious honeycomb shadows were observed, suggesting that the degree of pulmonary fibrosis was reduced, and the condition improved.

### 2.4. Reduced Expression of Inflammatory Factors after Interstitial Treatment

To detect the expression of related inflammatory factors in mice after model establishment and administration, we determined the levels of inflammatory factors in mouse plasma using ELISA. TGF-β1 plays an important role in lung fibrosis by promoting the proliferation and differentiation of fibroblasts into myofibroblasts, which further leads to the deposition of collagen and ultimately to fibrosis. Compared with the saline group (4961.1 pg/mL), TGF-β1 was significantly elevated in the plasma in animals of the BLM group, with plasma levels reaching 9147.2 pg/mL. After treatment, the BLM + HYAP group recovered to 4891.5 pg/mL, which was significantly lower than that of the BLM group (Figure 3B). At the onset of pulmonary fibrosis, M2 macrophages induce fibroblasts to secrete IL-6, which counteracts fibroblasts, induces their activation, and promotes the occurrence of pulmonary fibrosis. After BLM induction, the IL-6 content in the plasma of mice in the BLM group increased to 58.2 pg/mL. Compared with animals in the BLM + pirfenidone group (43.8 pg/mL), those in the BLM + HYAP group returned to normal levels (36.4 pg/mL), showing better therapeutic effects (Figure 3C).

### 2.5. Reduced Inflammatory Infiltration and Fibroblast Activation after Interstitial Treatment

**Figure 4:**
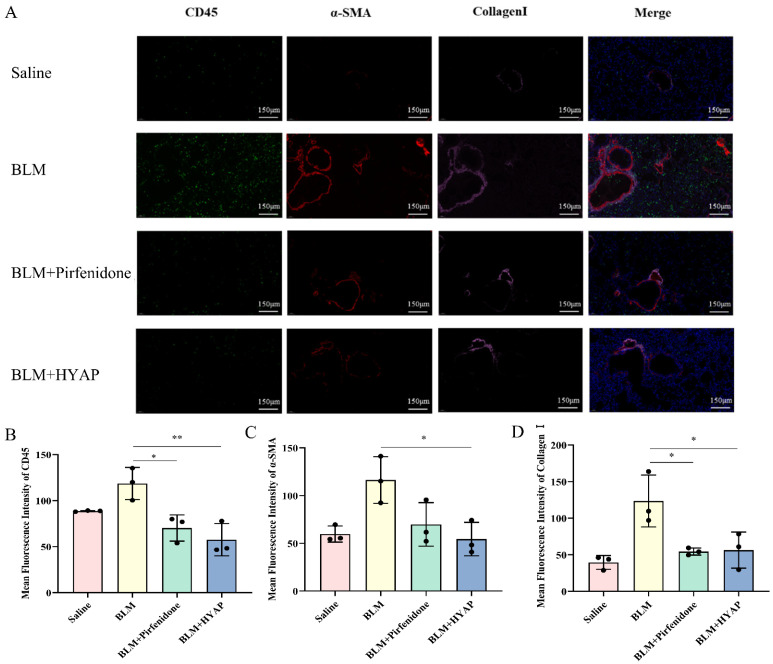
Immunofluorescence staining showing inflammatory infiltrates and fibroblast activation. (**A**) Immunofluorescence assay for CD45 (green), α-SMA (red), and collagen I (pink) expression in lungs (*n* = 3). Scale bar: 150 μm. (**B**–**D**) Expression of CD45, α-SMA, and collagen I in lungs after different treatments (*n* = 3). Fluorescence intensity was analyzed with Image J software. ** *p* < 0.01, * *p* < 0.05.

To further understand the inflammatory infiltration and fibroblast activation, we performed immunofluorescence staining (Figure 4A). Leukocytes were labeled with CD45 (green), activated fibroblasts were labeled with α-SMA (red), and collagen I expression was detected (pink).

CD45 was only slightly expressed in the saline group, whereas the BLM group showed a large amount of leukocyte interstitial infiltration. Compared with the BLM group, inflammatory infiltration in the BLM + pirfenidone and BLM + HYAP groups was significantly lower (Figure 4B). α-SMA exhibited low expression levels in the cytoplasm of smooth-muscle cells of larger blood vessels and bronchial walls in the lung tissue of mice in the saline-treated group. After BLM induction, α-SMA was strongly expressed in the lung interstitium, alveolar epithelium, and periphery of blood vessels in the BLM group, implying strong activation of myofibroblasts. Compared to the BLM group, α-SMA expression was significantly reduced in the BLM + pirfenidone and BLM + HYAP groups (Figure 4C). In the saline group, only a small amount of type I collagen expression was observed, which was consistent with the distribution of α-SMA and was mainly distributed around large airways. A large amount of collagen was deposited in the interstitium of the lungs in the BLM group. The expression of collagen I was also significantly reduced in the BLM + pirfenidone and BLM + HYAP groups compared with that in the BLM group (Figure 4D). The expression levels of CD45, α-SMA, and collagen I decreased in the BLM + HYAP group, indicating a reduction in lung inflammation in mice; moreover, the activation of fibroblasts was inhibited after HYAP treatment by ISI.

### 2.6. Inhibition of Pulmonary Fibrosis through the MAPK-p38 and TGF-β/Smad Signaling Pathways after Interstitial Treatment

**Figure 5:**
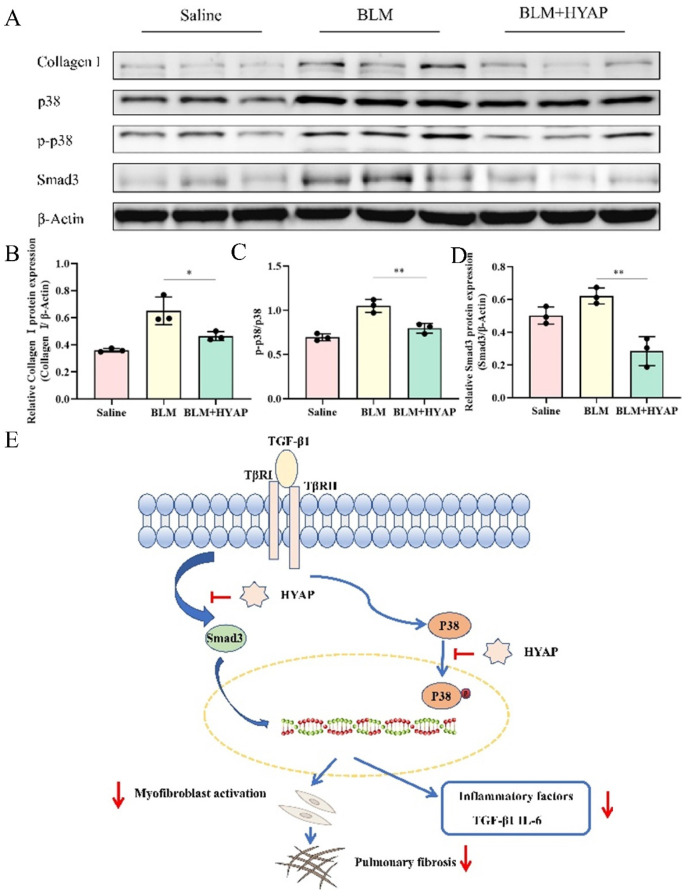
Expression of inflammation-related proteins after treatment. (**A**) Western blotting analysis of collagen I, p38, p-p38, and Smad3 expression levels in lung tissues after different treatments (*n* = 3). (**B**–**D**) Analysis of the expression of collagen I, p-p38/p38, and Smad3 in the lungs after different treatments (*n* = 3). Quantification was performed using Image J software. * *p* < 0.05, ** *p* < 0.01. (**E**) HYAP improved BLM-induced lung fibrosis by blocking the MAPK-p38 and TGF-β/Smad signaling pathways, thereby inhibiting secretion of related inflammatory factors and fibroblasts’ proliferation.

To further investigate the mechanism through which HYAP ameliorated pulmonary fibrosis, we investigated the signaling pathways associated with pulmonary fibrosis using Western blotting. Deposition of extracellular matrix is the most direct manifestation of pulmonary fibrosis; therefore, we first examined collagen deposition in the BLM and BLM + HYAP groups (Figure 5A). Consistent with the results discussed above, lung collagen expression was significantly upregulated in the BLM group, owing to pulmonary fibrosis, whereas collagen expression was significantly decreased in the BLM + HYAP group (Figure 5B).

The MAPK signaling pathway mainly includes ERK, JNK, and p38MAPK, which promote fibrosis by regulating inflammatory responses, mediating apoptosis, and participating in angiogenesis. This signaling pathway is triggered by a variety of extracellular stimuli. We analyzed the activation of p38 in mouse lung tissue using Western blotting (Figure 5A). There was no significant difference among the saline, BLM, and BLM + HYAP groups in terms of total p38 levels. We then examined the expression of p-p38, a protein downstream of MAPK-p38. Compared with the saline group, the expression of p-p38 was significantly higher in the BLM group, whereas the levels of p-p38 were significantly decreased in the BLM + HYAP group compared with the BLM group (Figure 5C), suggesting that HYAP inhibited the activation of p-p38, and thus blocked the MAPK-p38 signaling pathway, consequently reducing the inflammatory response and ameliorating pulmonary fibrosis.

We also determined the effects of the TGF-β/Smad signaling pathway on pulmonary fibrosis. TGF-β1 is an important factor in promoting pulmonary fibrosis, and the TGF-β/Smad signaling pathway plays an important regulatory role in the disease by promoting excessive deposition of extracellular matrix. Smad3 is an important protein in TGF-β1 intracellular signal transduction, which is the hub of the signaling pathway between the cell membrane and the nuclei and is the most critical and major signaling protein in the TGF-β1 signaling pathway. We found that the expression of Smad3 in the lung tissue of mice with BLM-induced pulmonary fibrosis was higher than that in the saline group (Figure 5D), and the expression of Smad3 in the BLM + HYAP group was lower than that in the BLM group (Figure 5D). Thus, these results suggest that the protective effect of HYAP on BLM-induced pulmonary fibrosis in mice is mediated by inhibition of the MAPK-p38 and TGF-β/Smad signaling pathways (Figure 5E).

## 3. Discussion

ISI is a novel drug delivery method for injecting drugs or nanoparticles into inter-stitial pathways using the ankle or carpal tunnel as ISI sites, which contain tendons, arteries, veins, and nerves. This transport pathway allows gold nanoparticles (AuNPs) to reach the central nervous system without crossing the blood–brain barrier, providing a new avenue for more effective treatment of central nervous system diseases [15]. Subsequently, the injection of malaria parasites through ISI better mimics the cutaneous phase of the natural malaria infection process, providing a new perspective on the mechanism of recurrence and enabling antimalarial treatment through this approach [16]. A disordered imbalance of macrophages and fibroblasts is the major regulator of pulmonary fibrosis. However, the origin of fibrotic macrophages and fibroblasts is not clear. Interstitial resident fibroblasts and macrophages are most likely fibrogenic precursor cells recruited from interstitial channels to the site of injury following stimulation of the lung interstitium. We introduced ISI into the treatment of pulmonary interstitial fibrosis to suppress cell recruitment at the injury site through interstitial therapy, which in turn suppresses inflammation and accelerates recovery.

HSYA has antioxidant, anti-inflammatory, and anti-cancer activities and a wide range of pharmacological effects on the cardiovascular, hematological, and nervous systems. However, it exhibits poor lipid solubility and low bioavailability when orally administered. We selected HSYA as the target drug for interstitial treatment of antifibrosis and prepared phytosomes to improve the lipid solubility of HSYA, thereby improving its bioavailability. The prepared HYAP had a uniform particle size, good stability, and a good, sustained effect. Subsequently, we compared the different administration concentrations. When the administration concentration of HYAP was 60 mg/kg, the degree of pulmonary fibrosis did not increase after 14 days of administration, and the alveolar structure tended to recover; however, the pharmacological effect was not obvious. The recovery of the alveolar structure increased significantly, and collagen accumulation decreased significantly, with increasing administration concentration. Combined with the clinical dosage, we selected 120 mg/kg for the treatment of the mouse model of pulmonary fibrosis.

Repair of lung tissue after injury is a complex phenomenon involving intricate mechanisms. In general, wound healing undergoes three dynamic and interrelated stages that overlap over time: inflammatory, proliferative, and remodeling phases of maturation and scar formation [25]. After repetitive injury, alveolar epithelial cells undergo damage, apoptosis, and capillary damage, leading to clot formation [26]. The initial source of cytokine in the wound is platelets present in the clot, which provide a variety of factors that stimulate the recruitment of neutrophils and macrophages [25]. Among these cytokines, TGF-β1 is a potent inducer of myofibroblast differentiation, which can also affect the balance between matrix metalloproteinases (MMPs) and their inhibitors (TIMPs), thereby favoring matrix deposition [27]. In the present study, the plasma levels of TGF-β1 increased significantly in the BLM group, whereas those of TGF-β1 were restored to normal levels in the BLM + HYAP group, thereby reducing the stimulation of fibroblasts and synthesis of extracellular matrix. The proliferative phase includes angiogenesis, which is essential for tissue repair because it provides vascular perfusion to the wound and delivers oxygen and nutrients, thereby promoting cell proliferation. On the other hand, fibroblasts in the injured tissue are activated and differentiate into myofibroblasts. Although α-SMA is also expressed in smooth-muscle cells and pericytes, it remains the most reliable phenotypic marker for myofibroblasts [28]. In the present study, using immunofluorescence, we confirmed the strong upregulation of α-SMA expression in the BLM group, whereas the expression levels of α-SMA in lung tissue decreased significantly in the BLM + HYAP group, suggesting that the proliferation and differentiation of fibroblasts were significantly inhibited by treatment with HYAP via ISI.

The role of the interstitium in pulmonary fibrosis has rarely been described. It has been shown that the subcutaneous interstitium is a major reservoir of fibroblasts, endothelial cells, and macrophages. In deep injury, fibroblasts in the subcutaneous interstitium direct their local complex matrix into the wound, coordinating with the coagulation cascade response and forming a prefabricated matrix that subsequently develops into mature scar tissue [29]. The interstitial microenvironment plays an important role in disease development. By adding wound fluid to the culture environment, the function of fibroblasts in the normal dermis may be altered to resemble fibroblasts in injured tissue [30]. Similarly, in the process of pulmonary fibrosis, fibroblasts and macrophages stored in the interstitium and interstitial fluid may play crucial roles in the occurrence and development of fibrosis. Therefore, it is important for us to try to identify an effective treatment for pulmonary fibrosis from an interstitial perspective. We attempted to identify an effective way to treat pulmonary fibrosis from an interstitial perspective. Using ISI in the upper limbs of mice, HYAP reached the corresponding organs through a non-vascular pathway, while inhibiting the proliferation and differentiation of fibroblasts in the interstitium by suppressing TGF-β/Smad signaling and further reducing collagen synthesis and secretion. HYAP entered the interstitial microenvironment through ISI and blocked the MAPK-p38 signaling pathway, possibly by inhibiting the activation of p38, and consequently, reducing the number of inflammatory factors in the interstitial fluid, thereby affecting fibroblast function and attenuating the progression of pulmonary fibrosis. However, whether ISI can play a more effective role in the treatment of pulmonary fibrosis needs to be further explored, but as far as the results are concerned, ISI has achieved good therapeutic results relative to oral positive drugs, proving the effectiveness of this treatment method.

## 4. Materials and Methods

### 4.1. Reagents

HSYA (C27H32O16; MW, 612.53; 98% Purity; Solarbio, Beijing, China), lecithin (Aladdin, Shanghai, China), bleomycin (BLM Biofeng, Shanghai, China), pirfenidone (Aladdin, Shanghai, China), ELISA kits (for TGF-β1 and IL-6; Cloud-Clone, Wuhan, China), 4% paraformaldehyde (Solarbio, Beijing, China), hematoxylin and eosin staining kit (beyotime, Shanghai, China), Weigert hematoxylin staining solution (Solarbio, Beijing, China), Ponceau S and Magenta staining solution (Solarbio, Beijing, China), phosphomolybdic acid solution (Solarbio, Beijing, China), Aniline blue solution (Solarbio, Beijing, China), DAPI (Solarbio, Beijing, China), RIPA buffer (Thermo Fisher Scientific, Waltham, USA), antibodies (for p38, p-p38, Smad3, collagen I, β-actin (Servicebio, Wuhan, China), anti-mouse IgG (Abcam, Cambridge, UK), anti-rabbit IgG (Abcam, Cambridge, UK)), BCA Protein Assay Reagent Kit (beyotime, Shanghai, China), and primary antibodies (CD45, α-SMA, and collagen I (Abcam, Cambridge, UK)), FITC-conjugated goat anti-rabbit IgG (Abcam, Cambridge, UK), CY3-conjugated goat anti-rat IgG (Abcam, Cambridge, UK), and CY5-conjugated goat anti-mouse IgG (Abcam, Cambridge, UK), were purchased.

### 4.2. Experimental Animals and Pulmonary Fibrosis Model Establishment

C57 BL/6J mice (male, 20 ± 2 g, 6 weeks old) were purchased from Beijing Vital River Laboratory Animal Technology (Beijing, China). C57 BL/6J mice were randomly divided into four groups: saline, BLM, BLM + pirfenidone, and BLM + HYAP groups. The experimental animals were anesthetized by inhalation of isoflurane, and the gas flow rate of the anesthesia machine was adjusted to 300 mL/min. After the anesthetic agent filled the anesthesia box, the animals were put into the anesthesia box with an anesthetic concentration of 3% and anesthesia time of 3 min. After the mice were completely anesthetized, the anesthesia mask was switched to continue the anesthesia on the experimental animals, followed by the next operation. The skin of the neck of the mice was dissected to expose the trachea, and 0.05 mL of BLM was injected at a dose of 5 mg/kg. An equal volume of saline was administered to the saline-treated group. This study was approved by the Institutional Animal Care and Use Committee (approval number: NCNST21-2104-0605).

### 4.3. Preparation of HYAP

The thin-film hydration method was used to prepare HYAP. HSYA powder and soybean lecithin were placed in a round-bottomed flask at a mass ratio of 1:3, ethanol was used as the solvent, and the mass concentration of HSYA was adjusted to 2 mg/kg. The samples were placed on a constant temperature magnetic stirrer and incubated at 40 °C for 2 h. After the reaction was complete, the unreacted precipitate was removed by suction filtration, and the organic solvent was removed by rotary evaporation under reduced pressure at 45 °C. An appropriate amount of chloroform and cholesterol (the mass ratio of phospholipid and cholesterol was 5:1) was added, shaken slightly to dissolve it, and any insoluble precipitates were removed via filtration. Continued rotary evaporation under reduced pressure at 45 °C formed a phytosome film on the bottom of the bottle. An appropriate amount of the saline was added to hydrate the phospholipid film for 30 min at 60 °C, followed by sonication for 15 min in a bath sonicator at 42 kHz and 100 W. The resulting phytosomes were subsequently extruded 10 times through a 100 nm polycarbonate porous membrane using an extruder (Avestin, Ottawa, ON, Canada).

### 4.4. Characterization of HYAP

#### 4.4.1. Size, Zeta-Potential, and Morphological Characterization of HYAP

The size, polydispersity index, and zeta-potential of HYAP were measured by DLS (Malvern, London, UK) in triplicate at 25 °C. The morphology was characterized by TEM (Hitachi, Tokyo, Japan). A drop of diluted HYAP was placed on a carbon-coated grid and air-dried, and a drop of 1% uranyl acetate was deposited onto the grid for 3 min. Excess uranyl acetate stain was removed from the edge with filter paper, dried overnight at room temperature, and used for TEM. 

#### 4.4.2. Measurement of HYAP Compounding Rate, Encapsulation Rate, and Release Rate In Vitro

Phospholipids and HYAP are soluble in chloroform, so the compounding rate of HSYA with phospholipids can be measured by dissolving HYAP in chloroform. The finished compounded solution was dissolved by rotary evaporation out of anhydrous ethanol, and the appropriate amount of chloroform was added. The free HSYA precipitated because it was insoluble in chloroform, the precipitate was removed, and the filtrate was broken by rotary evaporation with the addition of quantitative methanol ultrasonication. The content of the compound HSYA was measured at 403 nm using UV (Perkin Elmer, Boston, MA, USA).

A certain amount of HYAP squeezed by the extruder was placed in an ultrafiltration tube with a MWCO of 10,000 Da, centrifuged at 15,000 r/min for 30 min, and the nanoparticles were trapped in the inner tube, then the nanoparticles were collected and added to the quantitative methanol ultrasonication to break the emulsion.

The compounding rate and encapsulation rate were calculated using the following equations:

$$\mathrm{Compounding}\;\mathrm{Rate}\;\left(\%\right)=\frac{\mathrm{Total}\;\mathrm{HSYA}\;\left(w\right)-\mathrm{Free}\;\mathrm{HSYA}\;\left(w\right)}{\mathrm{Total}\;\mathrm{HSYA}\;\left(w\right)}\times 100$$

$$\mathrm{Encapsulation}\;\mathrm{Rate}\;\left(\%\right)=\frac{\mathrm{Total}\;\mathrm{HSYA}\;\mathrm{in}\;\mathrm{HYAP}\;\left(w\right)}{\mathrm{Total}\;\mathrm{HSYA}\;\left(w\right)}\times 100$$

The sustained release rate of the drug was determined by dialysis sampling and analyzed by UV. Using PBS as the release medium, stirring at 37 °C, 3 mL of HYAP was precisely measured in a pretreated dialysis bag. Samples (3 mL) were taken from the release medium at 0.5, 1, 2, 4, 6, 8, 10, 12, 24, 36, and 48 h and supplemented with equal volumes of PBS. The samples were filtered through a 0.22 μm microporous membrane and used for UV analysis. Absorbance values were measured at 403 nm and cumulative release rates were calculated.

#### 4.4.3. Ultraviolet Spectroscopy Analysis

The HSYA, phospholipids, physical mixture of HSYA and phospholipids, and HYAP were dissolved in methanol as the solvent and methanol was used as the blank, then the absorbance was measured in the wavelength range of 200–600 nm and the curves were plotted.

#### 4.4.4. Fourier Transform Infrared Spectroscopy Analysis

The mixture of HSYA and phospholipids, phospholipids, and HYAP with KBr was ground at 1:100, pressed into tablets, and then measured by FT-IR at wavelengths of 4000–500 cm^−1^.

### 4.5. Drug Treatment

Seven days after BLM injection, pulmonary fibrosis model mice were randomly divided into three groups of six mice each. The BLM + pirfenidone group was intragastrically treated with pirfenidone solution (200 mg/kg), daily for 14 days. The BLM + HYAP group was treated with HYAP in 0.1 mL of saline solution (120 mg/kg) by ISI in the right upper limb for 14 consecutive days, once a day. The chosen ISI injection point was the carpal tunnel of the wrist bone. The carpal tunnel is composed of the flexor retinaculum and carpal groove, inside which are the superficial and deep flexor tendons, flexor tendon sheath, flexor hallucis longus tendon and its sheath, and the median nerve and vessels running through it. The injection point was a convergent point for tendons, vessels, and nerve fibers connected to the fascia surrounding them. The saline and BLM groups were injected with an equal amount of saline via ISI in the right upper limb for 14 consecutive days, once a day.

### 4.6. CT Imaging Analysis

On day 14 of administration, mice were randomly selected from the saline, BLM, BLM + pirfenidone, and BLM + HYAP groups, and changes in lung structure during the establishment of fibrosis and drug treatment were recorded using CT in vivo imaging. CT was preheated for 15 min. The setting parameters were as follows: voltage, 90 kV; imaging current, 180 µA; real-time observation current, 180 µA; FOV, 24 mm; scanning program, 4.5 min; scanning angle, 360. The anesthesia machine was connected to an oxygen bottle, isoflurane was added, the gas source valve was adjusted at the front end of the oxygen flowmeter so that the output gas reached the required flow rate (300 mL/min), and the mice were placed in the anesthesia box. The anesthetized mice were fixed on the instrument cavity operating table, and the chamber was closed for scanning and analysis of the structure.

### 4.7. Histopathological Analysis

The CO_2_ asphyxiation method was adopted to execute the animal, whereby the animal was placed into the euthanasia box and CO_2_ was infused into the box at a rate of 20% of the volume of the box per minute replacement. It was confirmed that the animal was not moving and not breathing, the CO_2_ was turned off after the pupils were dilated, and then the animal was observed for 2 min to ensure that it was dead. Then, the left lower lung tissues were collected from each group on days 7 and 14 after administration and then fixed in 4% paraformaldehyde for 72 h. The samples were subsequently dehydrated using a series of gradients of ethanol, made transparent with xylene, and then embedded in paraffin. The samples were then cut into thin slices using an automated microtome, mounted on slides, and stored at room temperature for hematoxylin and eosin (HE) and Masson trichrome staining. After dewaxing, hydration, staining, dehydration, and sealing, pathological analysis was performed. Semi-quantitative analysis of pulmonary fibrosis was performed with reference to the Ashcroft scoring criteria, and the collagen volume fraction of Masson trichrome staining was calculated for different groups using Image J software.

### 4.8. Immunofluorescence

The largest lobe of the left lung was fixed with 4% paraformaldehyde, sliced using a frozen microtome, and stained with immunofluorescence. The sections were incubated overnight at 4 °C after adding the primary antibodies against CD45, α-SMA, and collagen I (1:50), and the slices were washed three times with TBST and incubated with FITC-labeled secondary antibody (1:200), CY3-labeled secondary antibody (1:300), and CY5-labeled secondary antibody (1:400) at room temperature for 3 h. The nuclei were stained with 4′,6-diamidino-2-phenylindole (DAPI) for 3 min, washed three times with TBST, dehydrated with gradient alcohol, made transparent with xylene, and sealed. Images were detected and collected by a slice scanner: DAPI glows blue by UV excitation wavelength 330–380 nm and emission wavelength 420 nm, FITC glows green by excitation wavelength 465–495 nm and emission wavelength 515–555 nm, CY3 glows red by excitation wavelength 510–560 nm and emission wavelength 590 nm, and CY5 glows pink by excitation wavelength 608–648 nm and emission wavelength 672–712 nm (CY5 was originally red, however to distinguish it from CY3, we set it to pink light). The staining results were analyzed using the Image J software.

### 4.9. ELISA

On day 14 of treatment, blood was collected from the ocular venous sinus and placed in blood collection tubes with EDTA. The upper plasma was collected after centrifugation at 4 °C and 3000 rpm for 5 min. ELISA kits were used to quantify the levels of TGF-β1 and IL-6 in the plasma according to the manufacturer’s instructions.

### 4.10. Western Blotting

A section (100 mg) of the lower lobe of the right lung was cut into small pieces on ice. After adding 1 mL of RIPA lysis solution and 10 μL of PMSF (100 mM), the sample was ground until there was no visible tissue and was left standing for 30 min or until complete lysis. The cells were centrifuged at 10,000× *g* at 4 °C for 10 min, and the supernatant was kept at −80 °C. Protein concentration was determined using the BCA method. Equal amounts of total protein were separated via sodium dodecyl sulfate-polyacrylamide gel electrophoresis and transferred to a nitrocellulose membrane using the semi-dry transfer method. After being sealed with 5% skim milk powder for 1 h at room temperature, the membrane was incubated with primary antibody diluted in phosphate-buffered saline overnight at 4 °C. After washing thrice with TBST, the proteins were incubated with horseradish peroxide-coupled IgG diluted in PBS at room temperature for 1 h. The proteins collagen I, p38, p-p38, and Smad3 were detected using enhanced chemiluminescence reagents, and the optical density values of the target bands were determined.

### 4.11. Statistical Analysis

Group comparisons were performed using the GraphPad Prism (version 9.0) software (GraphPad Software, San Diego, CA, USA). Ordinary one-way ANOVA was used for comparisons between more than two groups. Statistical significance was set at *p* < 0.05. * *p* < 0.05, ** *p* < 0.01, *** *p* < 0.001, and **** *p* < 0.0001.

## 5. Conclusions

In conclusion, our study showed that the delivery of HYAP via ISI ameliorated bleomycin-induced pulmonary fibrosis compared to oral positive drugs, which was associated with blocking the activation of p38 in the MAPK-p38 signaling pathway and reducing the content of Smad3 in the TGF-β/Smad signaling pathway, which in turn decreased the levels of relevant inflammatory factors and ameliorated the effects of pulmonary fibrosis. Whether interstitial administration can work better than traditional injection methods needs to be further explored. In the absence of an effective treatment, ISI does offer a reference strategy for the treatment of pulmonary fibrosis as a new potential method of drug delivery.

## Data Availability

Data is contained within the article.
